# A novel approach for sports injury risk prediction: based on time-series image encoding and deep learning

**DOI:** 10.3389/fphys.2023.1174525

**Published:** 2023-12-18

**Authors:** Xiaohong Ye, Yuanqi Huang, Zhanshuang Bai, Yukun Wang

**Affiliations:** ^1^ Chengyi College, Jimei University, Xiamen, China; ^2^ School of Physical Education and Sport Science, Fujian Normal University, Fuzhou, China; ^3^ School of Tourism and Sports Health, Hezhou University, Hezhou, China; ^4^ Institute of Sport Business, Loughborough University London, London, United Kingdom

**Keywords:** injury prevention, deep learning, time series, injury risk pattern, injury risk prediction

## Abstract

The rapid development of big data technology and artificial intelligence has provided a new perspective on sports injury prevention. Although data-driven algorithms have achieved some valuable results in the field of sports injury risk assessment, the lack of sufficient generalization of models and the inability to automate feature extraction have made it challenging to deploy research results in the real world. Therefore, this study attempts to build an injury risk prediction model using a combination of time-series image encoding and deep learning algorithms to address this issue better. This study used the time-series image encoding approach for feature construction to represent relationships between values at different moments, including Gramian Angular Summation Field (GASF), Gramian Angular Difference Field (GADF), Markov Transition Field (MTF), and Recurrence Plot (RP). Deep Convolutional Auto-Encoder (DCAE) learned the image-encoded data for representation to obtain features with good discrimination, and the classifier was performed using Deep Neural Network (DNN). The results from five repeated experiments show that the GASF-DCAE-DNN model is overall better in the training (AUC: 0.985 ± 0.001, Gmean: 0.930 ± 0.007, Sensitivity: 0.997 ± 0.003, Specificity: 0.868 ± 0.013) and test sets (AUC: 0.891 ± 0.026, Gmean: 0.830 ± 0.027, Sensitivity: 0.816 ± 0.039, Specificity: 0.845 ± 0.022), with good discriminative power, robustness, and generalization ability. Compared with the best model reported in the literature, the AUC, Gmean, Sensitivity, and Specificity of the GASF-DCAE-DNN model were higher by 23.9%, 27.5%, 39.7%, and 16.2%, respectively, which confirmed the validity and practicability of the model in injury risk prediction. In addition, differences in injury risk patterns between the training and test sets were identified through shapley additivity interpretation. It was also found that the training volume was an essential factor that affected injury risk prediction. The model proposed in this study provides a powerful injury risk prediction tool for future sports injury prevention practice.

## 1 Introduction

Running is one of the most popular sports in the world (Hulteen et al., 2017). Regular running can improve overall health by enhancing heart function, promoting blood circulation, and improving the respiratory and digestive systems. Additionally, running can enhance endurance and decrease the risk of cardiovascular disease (Hespanhol Júnior et al., 2015; Wewege et al., 2017). Despite the numerous benefits of running, it is crucial to acknowledge the inherent risk of injury that this physical activity entails. Research conducted by van Gent et al. (2007) demonstrates that the likelihood of injuries varies across running distances. More specifically, among individuals engaged in short-distance running of 15 km or less, reported incidences range from 14.3% to 44.7%. Conversely, long-distance runners participating in half-marathons or marathons demonstrate a greater exposure to injuries, with incidence rates ranging from 16.7% to 79.3%. Hespanhol Júnior et al. (2016) reported a running-related injury incidence rate of 7.7–17.8 per 1,000 h of running among athletes to support these findings. It is worth noting that approximately 80% of these running-related injuries are due to overuse, which means that most injury problems can be prevented by proper exercise management.

Identifying potential risk factors for sports injuries through training load monitoring and timely adjustment of the training program is vital for developing injury prevention strategies (Schwellnus et al., 2016; Soligard et al., 2016). Although there is much research evidence that excessive or sudden increases in training load are an important cause of injury (Gabbett, 2016; Gabbett et al., 2016), these phenomena were not found in the research reports by Suárez-Arrones et al. (2020). These conflicting research findings hinder the development of injury prevention strategies based on load management. Recent research reports suggest that misuse of statistical models may be the main reason for this spurious phenomenon. Bache-Mathiesen et al. (2021) analyzed the dose-response relationship between training load and injury risk by using multiple statistical models and found that nonlinear statistical models could fit the relationship sufficiently, while statistical models that assumed a linear association did not. The findings of certain studies support this view (Windt and Gabbett, 2016; Lathlean et al., 2017). Notably, some studies have found significant differences in the response patterns of individuals to the same training load, which implies that each individual’s tolerance, response, and recovery to training loads is highly personalized (Hubal et al., 2005; Buford et al., 2013). According to Simpson’s paradox in statistics, these differences may affect the statistical relationship between training load and injury risk to some extent, so the statistical regularity based on mass data cannot be better applied to different individuals (Tu et al., 2008). Furthermore, these studies often use binary variables as the outcome variables for injuries, which means that the outcome variable lacks some information related to the injuries (i.e., injury severity). Huang et al. (2022) pointed out that there are differences in the injury risk patterns corresponding to different levels of injury severity, which can lead to an inaccurate statistical relationship between training load and injury risk (The injury risk was coded in the form of a binary variable as the dependent variable). Thus, various factors complicate the relationship between training load and injury risk. Using statistical methods to explore the relationship between training load and injury can not meet the requirements of injury risk management in training practice. If a prediction tool for injury risk can be developed using training load monitoring and data-driven algorithms, it will be able to identify training load variation patterns related to injuries accurately, help reduce injury risks, and protect the body from harm.

With the rapid development of big data technologies and artificial intelligence, developing injury risk prediction tools based on data-driven algorithms has become possible. Fiscutean (2021) argues that standard methods for sports injury prevention rely on practitioners’ intuition and clinical experience, which may lead to incorrect clinical decisions due to cognitive biases. Rossi et al. (2022) believe that developing injury risk prediction models based on machine learning will help improve the efficiency of clinical diagnosis and promote the development of sports injury prevention strategies from empirical assessment to data-driven approaches. Currently, researchers have used some algorithms to build injury risk prediction models. These include Principal Component Analysis (PCA), Logistic Regression (LR), Decision Tree (DT), Linear Support Vector Machine (LSVM), and eXtreme Gradient Boosting (XGBoost). For example, Carey et al. (2018) created a hamstring injury prediction model (AUC: 0.76) for the Australian football club using training load data and the PCA-LR algorithm. Rossi (2017) used the LSVC-DT algorithm to build a non-contact lower limb injury prediction model for Italian professional men’s football players (Precision: 0.80, Recall: 0.76, F1 score: 0.78, AUC: 0.88). Lövdal et al. (2021) applied the Bagged-XGBoost algorithm in combination with the daily and weekly approaches to construct an injury risk prediction model for competitive runners (AUC_day approaches_: 0.724, AUC_week approaches_: 0.678). Although data-driven algorithms have produced some valuable results in the field of sports injury risk prediction, there are at least two problems that need to be addressed. First, the model lacks sufficient generalizability. According to a recent systematic review, regression is still the primary method for predicting injury risk, accounting for 60% of existing research reports (Bullock et al., 2022). The injury risk prediction based on regression can provide reasonable explanations, but this method is not sufficiently generalizable. In the real world, different injury risk patterns may lead to similar injury outcomes, which means that the correspondence between the same injury outcomes and risk patterns may not be unique (Bittencourt et al., 2016). If regression is used to solve this problem, the model may be underfitting or overfitting. It is worth noting that a few data samples are used to develop injury risk prediction models (median data sample size is 152, and median injury events are 57), which may lead to an optimistic estimate of model performance and the clinical value that the model can provide. There is a high risk of bias (Bullock et al., 2022). Some scholars argue that predictive performance is likely to deteriorate and uncertainty about clinical utility increases when the current study models are used in training practice with other groups of athletes (Carey et al., 2018; Bullock et al., 2022). Second, the feature construction strategy highly depends on the practitioner’s practical experience. The injury risk prediction based on training load is a multi-variable time-series classification problem. The feature construction of training load data is crucial in building an effective data-driven model. To our knowledge, current research has used chiefly sliding window algorithms for time-series data. For example, some scholars construct features by calculating statistical indicators such as the exponential weighted moving average and coefficient of variation of training load in the aggregate sliding window are training load markers for assessing injury risk (Colby et al., 2017; Carey et al., 2018; Rossi et al., 2022). Although these feature construction methods can effectively identify the injury risk, the constructed features are highly dependent on the knowledge and expert experience of the practitioner. Moreover, using these statistical features alone to find all the training load variation patterns associated with injury risk is insufficient, as individual tolerance, response, and recovery to training load are highly individualized (Collette et al., 2018). Lövdal et al. (2021) aggregated multivariate time-series data within a sliding window into one-dimensional feature vectors (i.e., one-dimensional feature vector representations of feature vectors at different moments) as input variables for the model. Although translating multivariate time-series data into a one-dimensional feature vector representation can capture the association between variables and injury risk at different moments, this feature construction approach loses the temporal and spatial correlation of time series data, which can lead to a reduction in the model’s predictive performance and make it difficult to apply the injury risk information extracted from the model to training practice. In recent years, modeling approaches that combine time series image encoding transformation with deep learning have been widely applied to address multivariate time series prediction and classification in industries and yielding promising results. The fundamental strategy behind this modeling approach is to convert a time series classification task into an image classification task by transforming one-dimensional time series data into two-dimensional images. This transformation enables automated feature extraction and powerful data processing capabilities, allowing the model to automatically capture potential patterns from dynamic and nonlinear time series data and make accurate predictions. However, this approach has yet to be extensively utilized in assessing injury risk in sports science and medicine. Consequently, there is an opportunity to leverage this modeling approach to develop a prediction model based on training load monitoring and capture potential patterns of training load variation associated with injury risk, which is critical for injury risk prevention.

This study aims to propose an injury risk prediction model based on time series image encoding and deep learning algorithms. Multiple time series image encoding, including Gramian Angular Summation Field (GASF), Gramian Angular Difference Field (GADF), Markov Transition Field (MTF), and Recurrence Plot (RP), were used to reconstruct the features for the dataset. Then, a Deep Convolutional Auto-Encoder (DCAE) is used to extract features from the image data to obtain a highly discriminatory representation of the features. Finally, the classifier was performed by applying Deep Neural Network (DNN) algorithms. The findings will help practitioners better understand the pattern of training load changes before sports injuries occur and predict sports injury risk by using data-driven models.

## 2 Materials and methods

### 2.1 Materials

The proposed methodology for this study was evaluated using a published dataset (Lövdal et al., 2021). The dataset comes from a 7-year training log (2012-2019) of a Dutch high-level running team and contains two datasets with the frequency of training days and the frequency of training weeks. Since runners’ endurance and recovery from training load is very individual, the training day frequency dataset was used in our study. According to Lövdal et al. (2021), this data set was processed with a time-sliding window. Each data sample consists of a vector of 70 variables (variables describe each data sample over the 7 days before the prediction day, and 10 variables describe each day).

### 2.2 Feature construction

#### 2.2.1 Feature vector reshape

**FIGURE 1:**
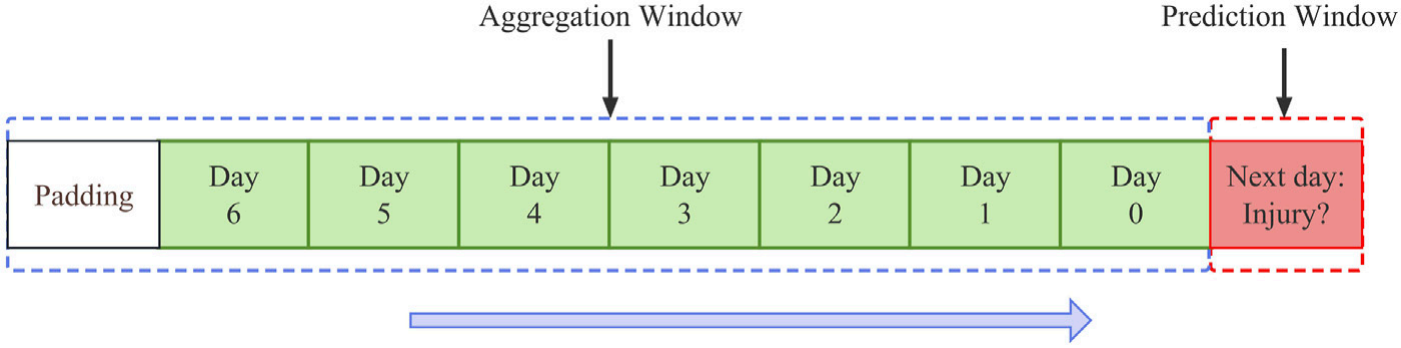
Feature vector structure: A data point is described by the features set during training for the 7 days before the prediction day. As the day approaches, a data point is 70 variables. This is because 10 variables describe each day. This study padded the original time series because the convolution layer needs to be the same size as the upsampling layer, both in the deep convolution auto-encoder.

Previous studies have reported significant differences in individual adaptability to different types of training loads (Collette et al., 2018), which means that different training load evaluation metrics may be sensitive to different types of injury risk. Compared to a single training load evaluation metric (e.g., running distance, rating of perceived exertion (sRPE), PlayerLoad™), including redundant and complementary training load information may help the system improve diagnostic precision and identify more injury risks (Buford et al., 2013). Therefore, this study reshapes the 70 variables in the raw data. The raw data was padded to satisfy the need for convolution (Figure 1). Each variable $X=\left\{x_{1},x_{2},\ldots ,x_{n}\right\},n=8$ was arranged in parallel and sorted by time to form a two-dimensional time-series dataset $D=\left\{X_{1},X_{2},\ldots ,X_{m}\right\},m=10$ of size N.

#### 2.2.2 Image encoding transform

**FIGURE 2:**
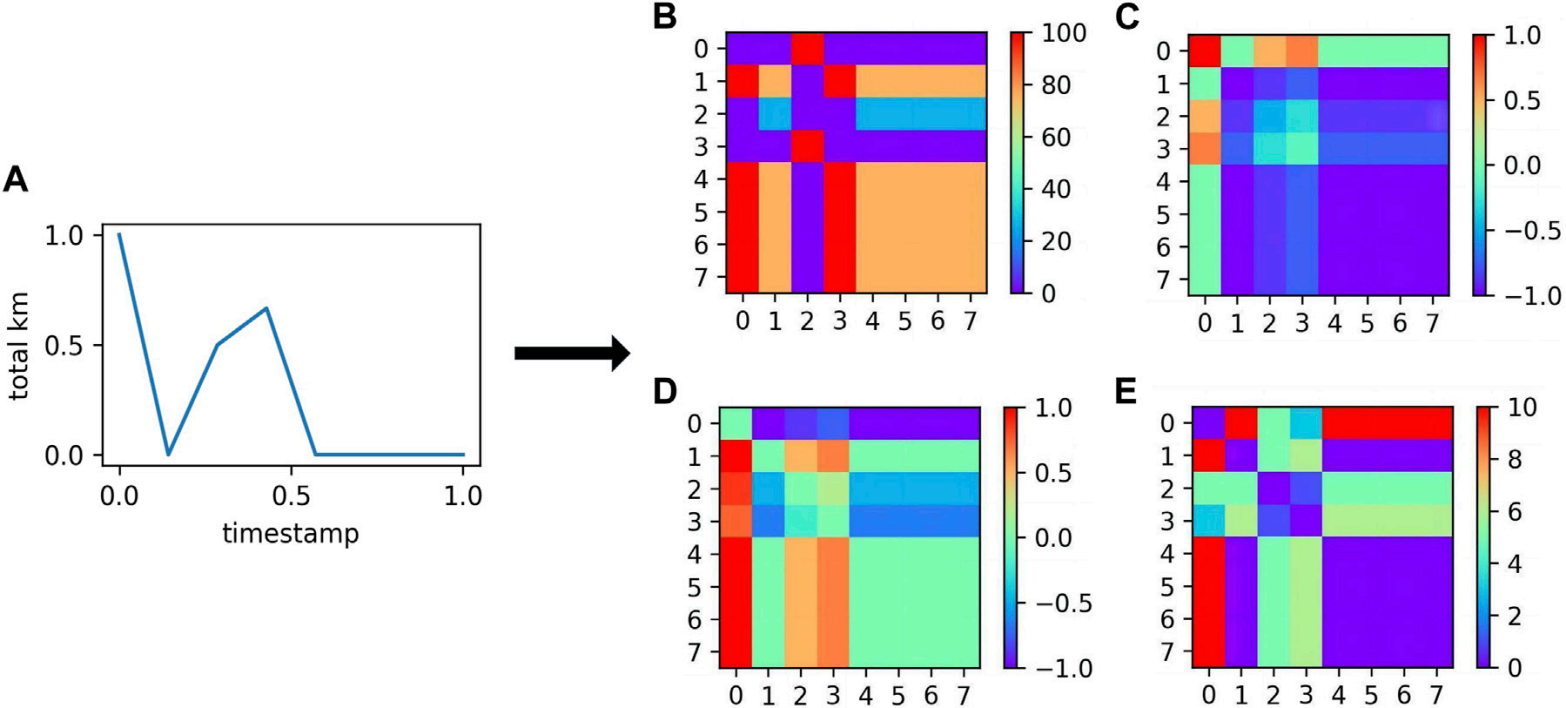
The feature graph of the normalized time-series transformation. **(A)** Time series after normalization; **(B)** Markov Transition Field; **(C)** Gramian Angular Summation Field; **(D)** Gramian Angular Difference Field; **(E)** Recurrence Plot.

Time series image encoding is a feature transformation method that converts information time series information into an image format with rich feature information, ensuring the completeness of the data. This study utilized three types of time series image encoding transformation methods: Gramian angular field transformation, Markov transition field transformation, and recurrence plot transformation, as shown in Figure 2.

**Gramian Angular Field Transformation.** Gramian Angular Field (GAF) is a method of encoding time-series images that preserves the time-series information and transforms it into an image format with rich feature information. It addresses the problem of time-series dependency while preserving the integrity of the information (Yang et al., 2019). It is implemented in the following steps:

First, all values of the univariate time series are scaled to the interval [0, 1] by the maximum-minimum normalization method (Eq. 1) to obtain the normalized variables $\tilde{X}=\left\{{\tilde{x}}_{1},{\tilde{x}}_{2},\ldots ,{\tilde{x}}_{n}\right\}$ .

$$\tilde{x}=\frac{x-x_{\min}}{x_{\max}-x_{\min}}$$ (1)

Next, the normalized values are encoded by using the arccos function (Eq. 2) and mapping the original time-series *X* to the polar coordinate using the timestamp encoding as the radius *r* (Eq. (3)). Where *θ* is the time-series value in polar coordinates for each observation. $t_{i}$ is the timestamp, and N is the total period of the time series.

$$\theta =\arccos\left({\tilde{x}}_{i}\right),where\;0\leq {\tilde{x}}_{i}\leq 1,{\tilde{x}}_{i}\in \tilde{X}$$ (2)

$$r=\frac{t_{i}}{N},t_{i}\in N$$ (3)

Finally, there are two types of images generated by GAF image encoding, the Gramian Angular Summation Field (GASF) and the Gramian Angular Difference Field (GADF), which are mathematically described in matrix format as Eqs 4, 5:

$$GASF=\left[\begin{array}{ccc} \cos\left(\theta_{1}+\theta_{1}\right) & \cdots & \cos\left(\theta_{1}+\theta_{n}\right)\\ \vdots & \ddots & \vdots \\ \cos\left(\theta_{n}+\theta_{1}\right) & \cdots & \cos\left(\theta_{n}+\theta_{n}\right) \end{array}\right]$$ (4)

$$GADF=\left[\begin{array}{ccc} \sin\left(\theta_{1}-\theta_{1}\right) & \cdots & \sin\left(\theta_{1}-\theta_{n}\right)\\ \vdots & \ddots & \vdots \\ \sin\left(\theta_{n}-\theta_{1}\right) & \cdots & \sin\left(\theta_{n}-\theta_{n}\right) \end{array}\right]$$ (5)

The GAF transformation can effectively preserve the information of the original time series, with the original information located in the main diagonal and the relationship between other time series reflected in other regions of the matrix.

**Markov Transition field Transformation.** The Markov Transition field (MTF) encodes time-series images using a Markov transition matrix (Han et al., 2021). The features extracted by this method can represent dynamic changes in time and frequency. This method includes the following steps: First, the time-series *X* is divided into Q bins (the Q was set to 5 in this study.) according to the range of values so that each $x_{i}\;\left(i\in \left\{1,2,\ldots ,n\right\}\right)$ can be mapped to its corresponding $q_{i}\;\left(i\in \left\{1,2,\ldots ,Q\right\}\right)$ . Second, the transition between $q_{j}$ is calculated in a first-order chain along each time step and a Markov transition matrix $T_{Q\times Q}$ is constructed (Eq. (6)).

$$\begin{array}{c} \begin{array}{c} W=\left[\begin{array}{c} \begin{array}{ccc} w_{11} & \ldots & w_{1Q} \end{array}\\ \begin{array}{ccc} w_{21} & \ldots & w_{2Q} \end{array}\\ \begin{array}{c} \begin{array}{ccc} \vdots & \ddots & \vdots \end{array}\\ \begin{array}{ccc} w_{Q1} & \ldots & w_{QQ} \end{array} \end{array} \end{array}\right] \end{array}\\ w_{i,j}=P\left(x_{t}\in q_{i}|x_{t-1}\in q_{j}\right) \end{array}$$ (6)

Where $\omega_{ij}\left(i,j\in \left\{1,2,\ldots ,Q\right\}\right)$ represents the probability that elements in $q_{j}$ are followed by $q_{i}$ elements. Finally, each probability is arranged chronologically to extend the Markov transition matrix, resulting in a Markov transformation field matrix *M* of size n×n . Where $M_{ij}\left(i,j\in \left\{1,2,\ldots ,n\right\}\right)$ is the probability that the bin corresponding to the time series $x_{i}$ is transferred to the bin corresponding to $x_{j}$ .

$$M=\left[\begin{array}{c} \begin{array}{ccc} \omega_{11}|x_{1}\in q_{i},x_{1}\in q_{j} & \ldots & \omega_{1n}|x_{1}\in q_{i},x_{n}\in q_{j} \end{array}\\ \begin{array}{ccc} \omega_{21}|x_{2}\in q_{i},x_{1}\in q_{j} & \ldots & \omega_{2n} \end{array}|x_{2}\in q_{i},x_{n}\in q_{j}\\ \begin{array}{c} \begin{array}{ccc} \vdots & \ddots & \vdots \end{array}\\ \begin{array}{ccc} \omega_{n1}|x_{n}\in q_{i},x_{1}\in q_{j} & \ldots & \omega_{nn}|x_{n}\in q_{i},x_{n}\in q_{j} \end{array} \end{array} \end{array}\right]$$ (7)

**Recurrence Plot Transformation.** Recurrence Plot (RP) is a visualization method of recurrence characteristics proposed by Eckmann et al. (1987). RP can obtain prior knowledge from the internal structure of time series, explain the similarity and information of time series, and analyze the predictability of signals. It is an important method for analyzing time series’ periodicity, chaos, and non-stationarity. The main idea of the RP is to reveal the movement of the trajectory from the current state to the previous state, which can be expressed as follows (Equations 8 and 9):

$$R_{i,j}=\theta \left(\varepsilon -\left\|{\vec{S}}_{l}-{\vec{S}}_{m}\right\|\right),\vec{S}\left(.\right)\in R^{n},l,m=1,2,\ldots ,K$$ (8)

$$\theta \left(x\right)=\left\{\begin{array}{c} 0\;x\leq 0\\ 1\;x>0 \end{array}\right.$$ (9)

Where *K* is the number of states of $\vec{S}$ and $\left\|{\vec{S}}_{l}-{\vec{S}}_{m}\right\|$ means the closeness of the two vectors ${\vec{S}}_{l}$ and ${\vec{S}}_{m}$ in the phase space. ε is the threshold of distance and $\theta \left(.\right)$ is the Heaviside function. The two vectors are close to each other, or recurrence occurs when $\left\|{\vec{S}}_{l}-{\vec{S}}_{m}\right\|<\varepsilon$ . When $R_{i,j}=1$ , black points are used to indicate the state of recursion, and when $R_{i,j}=$ 0, white points indicate that no recursion is occurring. This allows a two-dimensional recursive plot to be created. This study uses an actual sample to show the transformation process of time series and matrix by different methods, which can be found in Supplementary File S1.

### 2.3 Data processing

**Normalization.** Because of the significant differences in the stimulus-response of individual runners to different training loads, the data from individual runners were processed with minimum-maximum normalization transformation (Eq. (1)) so that the model could identify similar injury risk patterns through cross-sectional comparison.

**Multiple Resampling.** The dataset is highly imbalanced, which may cause the classifier to identify a minority class poorly. Therefore, this study uses multiple resampling to process the dataset to reduce this imbalance. The procedure:

Step 1: Balanced sampling for each athlete dataset. There are different injury events in each runner’s raw data (i.e., a biased dataset). If the original dataset is used directly to train the model, it may result in the model only identifying injury events for runners with a higher risk of injury. Following Lövdal et al. (2021), this study randomly selects an equal number of injured and uninjured samples from all subsets of runners in the training set to generate an unbiased and balanced dataset (i.e., an equal number of injured and uninjured samples for all runners), to avoid this problem.

Step 2: Unbalanced sampling of the unbiased and balanced dataset. The unbiased and balanced dataset has many duplicate samples, and the injury distribution does not match the real-world situation, which can easily make the model training slower and overfit. For this reason, the unbiased and balanced dataset was unbalanced by a fixed number of injury event samples and sampling ratio to produce a subset of unbiased unbalanced data, where the number of injury event samples was set to 650 (i.e., The number of runners is multiplied by the average number of runner injury events) and the sampling ratio was controlled to 0.136 (i.e., Ten times the number of minority samples divided by the number of majority samples)

Step 3: The unbiased and unbalanced subset is synthetically sampled using the SMOTETomek algorithm (which is a combination of Synthetic Minority Oversampling Technique and Tomek Links Undersampling). This study processed the tomek link (i.e., sample points A and B from two different classes are nearest neighbors) in the feature space to improve the model’s generalization ability to identify injury events (Chawla et al., 2002). Because the number of minority class and majority class samples in the dataset were very different, only the sample points in the Tomek link that belonged to the majority class were removed in this study. The minority samples were then synthetically sampled using the Synthetic Minority Over-sampling TEchnique (SMOTE) to generate the training set for model construction. SMOTE is an improved technique based on random oversampling proposed by Chawla et al. (2002). It can effectively solve the problem of poor generalization due to random oversampling. The algorithm determines the k-nearest neighbors of each minority class sample by calculating the Euclidean distance from each minority class sample to all minority class samples. The sampling ratio is set according to the sample balance rate. Some samples are selected from the k nearest neighbors of each minority class sample by generating a new sample (Eq. (10)).

$$d_{new}=d+rand\left(0,1\right)*\left(d-d_{n}\right)$$ (10)

### 2.4 Model architecture

**Feature Representation.** This study used deep convolutional auto-encoder (DCAE) for feature representation extraction to solve the problem of big data and limited *a priori* knowledge. DCAE is an auto-encoder consisting of multiple convolutional, pooling, compression, and hidden layers. This architecture gives the model better representation capabilities and more robust features. DCAE is implemented through a symmetric encoding and decoding structure for the data reconstruction process, which is described as follows (Equations 11 and 12):

$$h\left(x\right)=\sigma_{e}\left(w∙x+b\right)$$ (11)

$$x^{\prime }=\sigma_{d}\left(w^{\prime }∙h\left(x\right)+b^{\prime }\right)$$ (12)

Where w **,** b are the encoding weights and biases, $w^{\prime }$ **,** $b^{\prime }$ are the decoding weights and biases, and $\sigma_{e}$ , $\sigma_{d}$ are the non-linear transformations during encoding and decoding, respectively. The adadelta optimizer is used to optimize DCAE by minimizing the Mean Squared Error (MSE) of $x_{i}$ and ${\hat{x}}_{i}$ . The epoch of the model training process was set to 100 and the batch to 512. The initial learning rate of the adadelta optimizer was set to 1.0 with a decay rate of 0.95. Its reconstruction error function is expressed as follows (Eq. (13)):

$$L_{mse}=\frac{1}{n}\sum_{i=1}^{n}{\left({\hat{x}}_{i}-x_{i}\right)}^{2}$$ (13)

**FIGURE 3:**
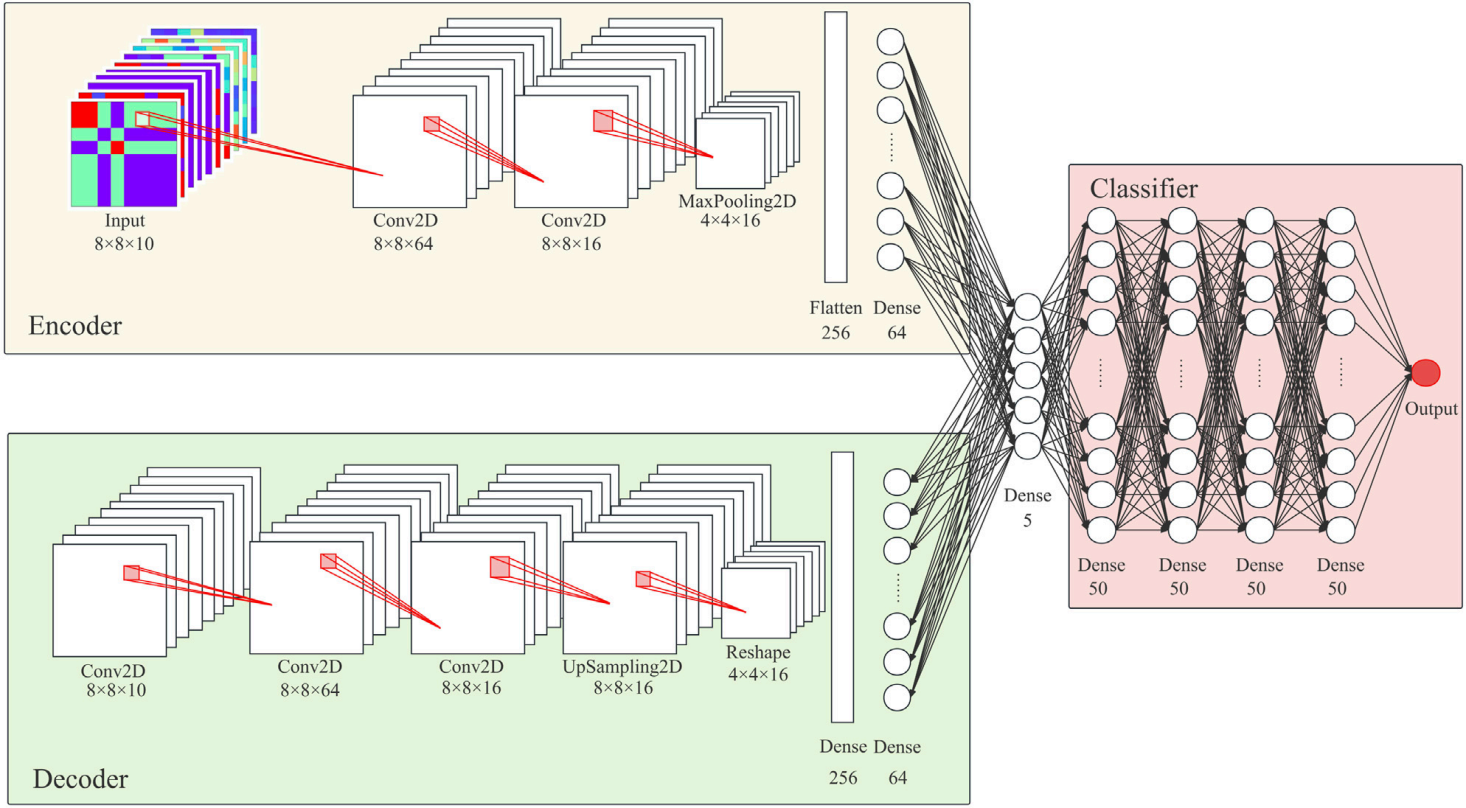
Model Architecture: The model proposed in this study consists of a feature representation module based on a deep convolution self-encoder and a classifier module based on a deep neural network.

This study applies batch normalization and dropout to each hidden layer in DCAE and uses Scaled Exponential Linear Units (SELU) as the activation function (Hu et al., 2020). The output layer of the decoder selects the hyperbolic tangent function as the activation function. Figure 3 shows the number of neuron units per layer.

**Classifier.** According to the general opinion in sports science, the relationship between training loads and injury risk is non-linear (Bache-Mathiesen et al., 2021). Therefore, this study considers that using a deep neural network to construct a classifier would benefit training load-based injury risk management. The model will predict whether an injury will occur the following day using the representational features obtained from the DCAE as input. The model’s architecture consists of an input layer, four hidden layers, and an output layer. Each hidden layer contains 50 neurons, and each is subjected to batch normalization and dropout, coupled with the use of SELU as the activation function. The output layer of the model uses the sigmoid function as the activation function. Since the dataset has an extreme class imbalance distribution, this study uses the adadelta optimizer and introduces the Focal loss function to improve the model’s training process. The epoch of the model training process was set to 100 and the batch to 512. The initial learning rate of the adadelta optimizer was set to 1.0 with a decay rate of 0.95. Focal Loss is a loss function proposed by Lin et al. (2020) to solve the class imbalance problem, which allows the model to focus more on the hard-to-classify samples during training by reducing the weights of the easy-to-classify samples. Introducing a balance coefficient α and a focus coefficient γ into a single cross-entropy loss function to adjust the loss weights of different class samples makes the model focus more on the minority class samples that are difficult to classify. The loss function is shown in Eq. (14).

$$L_{FL}=\left\{\begin{array}{c} -\alpha {\left(1-{\hat{y}}_{n}\right)}^{\gamma }\log{\hat{y}}_{n}\;{if\;\hat{\;y}}_{n}=1\\ -\left(1-\alpha \right){{\hat{y}}_{n}}^{\gamma }\log\left(1-{\hat{y}}_{n}\right)\;if\;{\hat{y}}_{n}=0\; \end{array}\right.$$ (14)

In Eq. (14), ${\hat{y}}_{n}$ is the probability that the predicted sample category is 1, and $1‐{\hat{y}}_{n}$ is the probability that the predicted sample category is 0. The α coefficient is used to adjust the weight of different categories of samples in the loss function. The loss weights of the minority class samples will be increased when $\alpha \in \left(0.5,1\right)$ . The focus factor γ is used to adjust the loss weights of the easy and hard-to-classify samples. If the value of γ becomes greater, the loss value of the easy-to-classify samples will be smaller. This study’s tuning process for *α* and *γ* was based on empirical. The α for the optimal model was approximately 0.986 (i.e., 1 minus the ratio of minority samples to the total sample), while γ was set to 3.5. All of the model parameters are shown in the Supplementary File S2.

### 2.5 Model training, validation, and testing

**FIGURE 4:**
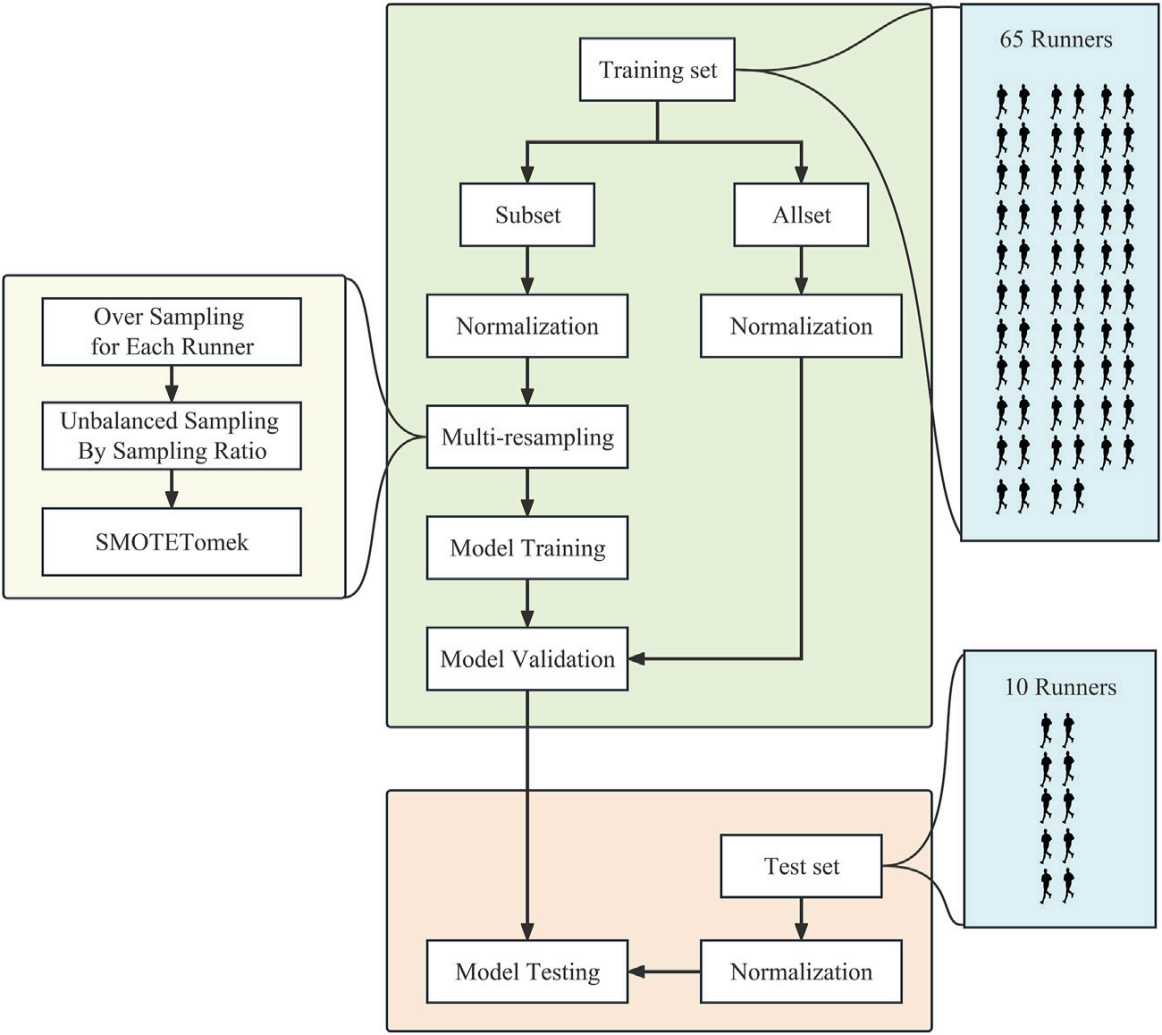
The flowchart for model training, validation, and testing.

This study evaluated the model performance’s internal and external validity using hold-out methods, which can provide discriminative power of predictive models regarding sports risk injuries. The training set consisted of data from 64 athletes, containing 39,189 uninjured and 533 injured samples, while the test set consisted of data from 10 athletes, containing 2,994 uninjured and 50 injured samples. Following Lövdal et al. (2021), this study randomly selected part of the dataset in a training set for model fitting. It validated the model on the whole training set to assess the internal validity of the model (Figure 4). The model’s training, validation, and testing procedure was repeated five times with consistent parameters in each experiment.

This study’s training set was divided by random sampling. Because of the uneven distribution characteristics of random seeds that may lead to additional sampling bias, our study used the best discrepancy sequence suggested by Guo et al. (2019) to randomize for each trial. The area under the receiver operating characteristic curve (AUC), sensitivity, specificity, and geometric mean (Gmean) were chosen as the metrics for evaluating model performance. The calculation equation is described as follows (Eqs (15)–(17):

$$Sensitivity=\frac{TP}{TP+FN}$$ (15)

$$S\;pecificity=\frac{TN}{TN+FP}$$ (16)

$$Gmean=\sqrt{Sensitivity\times Specificity}$$ (17)

TP , FP , TN , and FN indicate true positives, false positives, true negatives, and false negatives. Sensitivity and specificity, also known as true positive and negative rates, are significant evaluation indicators in medical clinical diagnosis. Suppose a predictive model has lower sensitivity or specificity. In that case, it implies that the model has a higher rate of misdiagnosis or underdiagnosis, which can prevent its deployment in real-world applications. Gmean is an overall metric that incorporates sensitivity and specificity. It effectively reflects the overall classification performance of a model on both majority and minority class samples. A higher Gmean is achieved when both sensitivity and specificity are high.

### 2.6 Injury risk pattern analysis

The interpretation of the model’s decisions is particularly important for training practice, which should provide the practitioner with a full, logical explanation of the decision, which can help coaches and team doctors develop good training programs and make targeted interventions. Deep learning is a black box, meaning the variable’s importance and working mechanism cannot be as straightforward as a regression. Therefore, this study uses SHapley Additive exPlanations (SHAP) for attribution analysis of feature representation and classifier (Nohara et al., 2019). The absolute weights of each variable were calculated from Eq. (18). The Python 3.6 programming environment was used to train, test, validate, and analyze the models.

$$Importance=\frac{\sum_{i=1}^{N}\left({\left|SHAP\right|}_{i}\right)}{N}$$ (18)

### 2.7 Statistical analysis

This study uses Welch’s analysis of variance (ANOVA) to analyze differences and the Games-Howell Post-Hoc Test for multiple comparisons and reporting Mean Difference (MD). All hypothesis tests were conducted using two-sided hypothesis tests, setting α in the hypothesis test to 0.05 and considering where *p* > 0.05 as not significant and *p* < 0.05 as significant.

## 3 Results

### 3.1 Injury prediction

**FIGURE 5:**
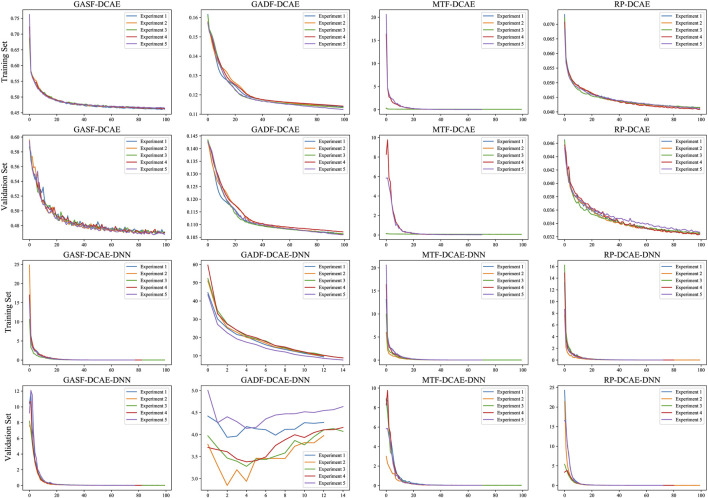
Loss curves for feature representation modules and classifiers.

As shown in Figure 5, the loss curves of the feature representation module (time-series image encoding - deep convolutional auto-encoder) tended to be the same overall in the training and test sets, indicating that the deep convolutional auto-encoder was able to fit the data well. The GADF-DCAE-DNN model showed significant overfitting in the classifier, indicating that the GADF-DCAE-DNN has poor generalization ability. The loss curves of the other models tended to be the same overall in the training and test sets, implying that they fit the data well.

The training and test sets assessed the models’ internal and external validity (Table 1). It should be pointed out that the model construction program in this study is inconsistent with the internal validity evaluation method proposed by Lovdal et al., so the internal validity evaluation of Bag-XGBoost proposed by Lovdal et al. is not included in Table 1. The results showed that the RP-DCAE-DNN performed best in the training set, with the highest average-AUC (0.998), average-Gmean (0.972), average-Sensitivity (0.998), and average-Specificity (0.947). The average-Sensitivity of the MTF-DCAE-DNN and the GASF-DCAE-DNN is second only to the RP-DCAE-DNN, which are 0.998 and 0.997, respectively. At the same time, the average-Specificity is significantly lower than the RP-DCAE-DNN, which are 5.81% (MD = −0.054, *p* = 0.010) and 8.34% (MD = −0.079, *p* < 0.001) lower respectively. It shows that the RP-DCAE-DNN has the best internal validity.

**TABLE 1:** Performance evaluation results of models in the training and test set.

Model	AUC		Gmean		Sensitivity		Specificity	
	Training set	Test set	Training set	Test set	Training set	Test set	Training set	Test set
Bag-XGBoost	**—**	0.719 ± 0.007	**—**	0.651 ± 0.021	**—**	0.584 ± 0.054	**—**	0.727 ± 0.027
RP-DCAE-DNN	**0.998 ± 0.001**	0.889 ± 0.016	**0.972 ± 0.008**	**0.842 ± 0.015**	**0.998 ± 0.001**	**0.920 ± 0.028**	**0.947 ± 0.015**	0.772 ± 0.043
MTF-DCAE-DNN	0.986 ± 0.001	0.759 ± 0.041	0.944 ± 0.009	0.682 ± 0.024	0.998 ± 0.002	0.644 ± 0.046	0.892 ± 0.018	0.724 ± 0.055
GADF-DCAE-DNN	0.646 ± 0.019	0.594 ± 0.070	0.537 ± 0.044	0.498 ± 0.104	0.376 ± 0.084	0.340 ± 0.154	0.783 ± 0.067	0.772 ± 0.063
GASF-DCAE-DNN	0.985 ± 0.001	**0.891 ± 0.026**	0.930 ± 0.007	0.830 ± 0.027	0.997 ± 0.003	0.816 ± 0.039	0.868 ± 0.013	**0.845 ± 0.022**

The bold value for the model performed best in the training or test set.

This study found that GASF-DCAE-DNN has the best average-AUC (0.891) and average-Specificity (0.845) in the test set. Although the average-Gmean (0.830) of the GASF-DCAE-DNN was 1.43% (MD = −0.012, *p* = 0.966) lower than that of the RP-DCAE-DNN, this difference was not significant. It is worth noting that the standard deviation of the performance metrics of the GASF-DCAE-DNN is minimal, which indicates that the GASF-DCAE-DNN can perform better in prediction while being less sensitive to sampling bias. In addition, it is essential to note that RP-DCAE-DNN has the best average-Sensitivity (0.920), which indicates that RP-DCAE-DNN can identify injured samples well. However, the average-Specificity of the RP-DCAE-DNN was only 0.772, which means that 23.8% of the not-injured samples were misclassified as injured. Overall, the GASF-DCAE-DNN has good discrimination, robustness, and generalization ability, which makes the model more appropriate for application in injury risk prediction for runners.

This study compares the performance of the best model with the injury risk prediction model based on Bag-XGBoost proposed by Lövdal et al. in the test set. It was found that the GASF-DCAE-DNN significantly outperformed the Bag-XGBoost, with an improvement of 23.9% (MD = −0.172, *p* < 0.001), 27.5% (MD = −0.180, *p* < 0.001), 39.7% (MD = −0.232, *p* < 0.001) and 16.2% (MD = −0.118, *p* < 0.001) in AUC, Gmean, Sensitivity, and Specificity, respectively. This result implies that the best model proposed in this study can diagnose more injury risks with fewer misdiagnoses.

### 3.2 Feature importance and risk pattern

**FIGURE 6:**
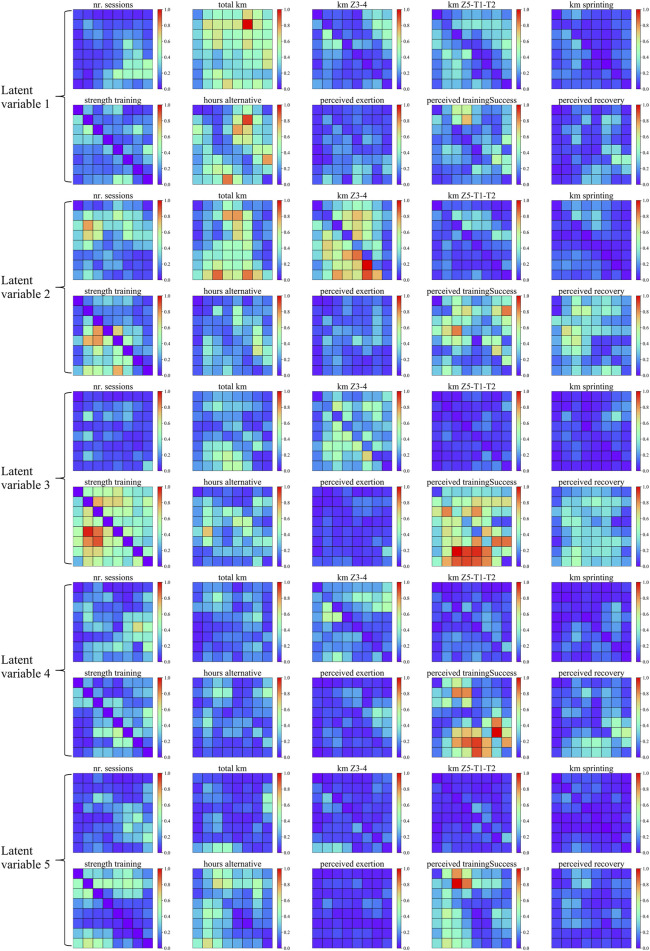
The feature heat map corresponds to the latent variables. The image data in the figure are all derived from the original time series after the Gramian Angular Summation Field transformation. The relative importance is normalized for presentation in this study to see the relatively important variables better.

SHAP was used to calculate the variable importance to latent variables to understand the meaning of latent variables after dimension reduction. Figure 6 shows a heatmap of variable importance for the latent variable. “total km” and “alternative hours” have greater variable importance for latent variable 1, indicating that latent variable 1 may represent training volume (including running and cross-training). “km Z3-4″, “total km”, “strength training”, and “perceived training success” had greater variable importance for latent variable 2, indicating that latent variable 2 may represent the volume of anaerobic intensity training (including medium to high-intensity running and strength training). “strength training” and “perceived training success” have higher variable importance for latent variable 3, meaning that latent variable 3 may represent strength training. “perceived training success” has greater variable importance for latent variables 4 and 5, meaning that latent variables 4 and 5 relate to what athletes thought about training.

**FIGURE 7:**
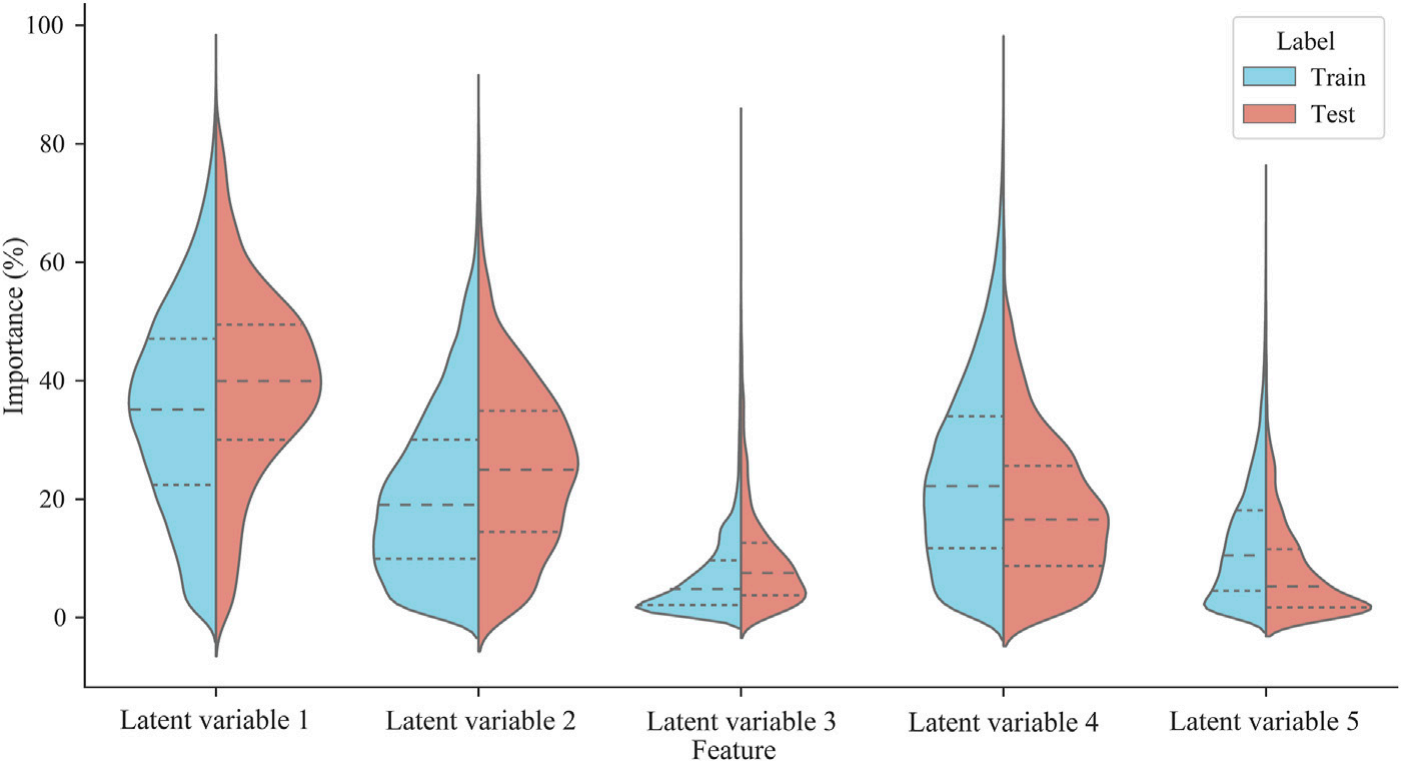
The relative importance of latent variables in training and test sets. The dotted lines in the figure indicate the upper quartile, median, and lower quartile.

Through feature attribution of model decisions, it was found that although latent variable 1, latent variable 2, and latent variable 4 had high relative importance, their relative importance significantly differed in the proportion of the training set and test set (*p* < 0.01). As shown in Figure 7, there are significant differences in the relative importance of latent variables 1 (MD = −0.043, *p* < 0.001), latent variables 2 (MD = −0.041, *p* < 0.001), latent variables 3 (MD = −0.026, *p* < 0.001), latent variables 4 (MD = 0.061, *p* < 0.001) and latent variables 5 (MD = 0.049, *p* < 0.001) in both training and test sets. Among them, the relative importance of latent variable 1, latent variable 2, and latent variable 4 in the training set were 35.0%, 21.2%, and 24.1%, respectively. Latent variable 1, latent variable 2, and latent variable 4 in the test set were 39.3%, 25.2%, and 18.0%, respectively. The results suggest that there may be differences in injury risk patterns between the training set and the test set. Among them, the relative importance of latent variable 1 ranks first in the training and test set, which means that the training volume is a significant predictor of the model’s performance in predicting the runner’s injury risk.

## 4 Discussion

This study constructed an injury risk prediction model based on time series image encoding and deep learning algorithms by using training load monitoring data. To a certain extent, this research can provide the necessary reference for developing injury risk prediction tools based on training load monitoring and data-driven algorithms. There are three main findings: firstly, combining time-series image encoding with deep learning feature representation can extract latent information distinguishing injured and non-injured samples. Secondly, it was found that the model constructed using GASF combined with deep learning has discrimination, robustness, and generalization capabilities, which is significantly better than the existing model of injury risk prediction for runners. Finally, the model’s feature attribution analysis identified that training volume seems to be a significant predictor of runner injury risk.

### 4.1 Time series image encoding-feature representations can extract latent feature related to injury from training load

The relationship between training load and injury risk is complicated, and extracting information that distinguishes injury risk from training load data is difficult. Several researchers have conducted a series of studies that sought to extract features associated with injury risk from training load data. For example, Foster, Gabbett, and others attempted to predict injury risk using statistics indicators (e.g., coefficient of variation and exponential weighted moving average) that quantify the variability and accumulation of training load (Foster et al., 1995; Gabbett et al., 2016). Although the method provides several practical metrics, the reliability and validity of the method will significantly be affected by individual differences and the training load quantification method. For instance, Gabbett et al. (2016) found an association between exponentially weighted moving averages of training load and injury risk that was not found in some research (Suárez-Arrones et al., 2020). It is important to note that most studies use a single evaluation method to quantify training loads, such as distance, session RPE, and other indicators, which may lead to statistical indicators of training load associated with injury risk that are not personalized. McLean et al. (2010) investigated the neuromuscular, endocrine, and perceptual responses of elite rugby league players in different recovery periods and found that individual responses to training load were highly individualized. Furthermore, adaptation and fatigue to training load are associated with the accumulation, and using multivariate time-series data prediction methods would ignore this relationship. Thus, a complicated feature construction method and automatic feature representation extraction are necessary. To improve this shortcoming, we have attempted various time-series image encoding transformation methods for the feature reconstruction of multivariate time-series data, which add representations of relationships between values at different moments. Time series image encoding represents a methodology for converting time series data into image format. Compared to traditional time-series data representations, this technique captures a greater abundance of details and features by retaining the data’s time-series dependencies and inherent structural features. This image-based representation facilitates a more intuitive and comprehensive visualization of the trends and patterns underlying temporal changes in the data.

### 4.2 Advantages of deep learning in injury risk prediction

Although numerous scholars have used various statistical models and machine learning to develop predictive models for sports injury risk and provide valuable findings, the models developed have been poor in accuracy, generalization, and stability, preventing the models from being deployed in training practice. To our knowledge, the reasons for this problem can be attributed to three aspects. Firstly, from a sports science perspective, there is not always a direct correlation between training load and injury risk. Instead, it may indirectly influence injury risk by regulating the adaptability and physical fitness of the human body (de Leeuw et al., 2021). More specifically, the training adaptability of the human body is a continuous process in which changes in intrinsic risk factors such as previous injuries, age, sleep, biomechanics, and psychological factors can influence the tolerance to training load. Similarly, the effects of training load can affect these intrinsic risk factors. As a result, the relationship between training load and injury risk varies significantly between individuals, obscuring the numerical relationship between the two (Windt et al., 2018). Secondly, from the perspective of model selection, parametric models have been widely used in sports injury prediction modeling, which has simplicity, reliability, and interpretability advantages. However, the poor generalization of model coefficients and many assumptions in the models have led to poor performance in injury risk prediction (Ruddy et al., 2019). Finally, from a mathematical perspective, injury risk patterns are theoretically unique in the real world. However, they suffer from two significant limitations - insufficient *a priori* knowledge and limited information on risk factors - which make it impossible to find information on injury risk patterns and predict injury risk using an analytical solution. Based on the above three perspectives, we suggest that deep learning is more applicable, which has the advantages of high accuracy, powerful representation, and the ability to capture complex pattern information in the data. This study has used deep learning to build injury risk prediction models based on training load data and achieve good prediction performance. By comparing the model’s prediction performance in the training and test sets, it was found that the RP-DCAE-DNN had the best AUC, Gmean, and Sensitivity, implying that this model could predict the injury risk well. However, this model’s specificity was low, which may lead to the model being abandoned due to the large number of misdiagnoses in the application. It is noted that the GASF-DCAE-DNN has overall better discrimination, robustness, and generalization of the prediction performance in both the training and test sets, even though the AUC, Gmean, and Sensitivity of the GASF-DCAE-DNN are not the best. These are two reasons that injury does not always occur due to conditions with injury risk and that injury does not always happen caused by training load. Therefore, GASF-DCAE-DNN is the best prediction model in this study.

### 4.3 Training volume seems to be a significant predictor of injury risk

Injury risk prediction needs not only to predict the occurrence of sports injuries but also to identify essential features that predict injury risk (Ruddy et al., 2019). This study used the SHAP approach to analyze the feature representation module’s and classifier’s variable importance. Then, it was found that training volume may be a significant predictor for injury risk prediction, followed by training volume at the anaerobic intensity and what athletes thought about training. Previous studies have shown that training volume is strongly associated with injury risk. Colby et al. (2014) found that long-term cumulative and sprint distances were positively associated with pre-season injury risk in 46 elite Australian football players. Malone et al. (2018) found that higher training loads were associated with a significantly higher risk of injury in 48 professional football players. O'Keeffe et al. (2019) identified short-term workload, training load monotony, and the amount of weekly load change as risk factors for injury in 97 male youth athletes in Gaelic football. In contrast to previous investigations, the dataset used in the present study incorporates a comprehensive set of multi-dimensional training load assessment metrics, including distance, distance covered under different physiological states, training logs, and subjective perception of exertion. By incorporating these diverse variables, our study facilitates a multifaceted exploration of the associations between training load and injury risk. This approach enables a more holistic understanding of the relationships between training load and injury risk, offering valuable insights from multiple perspectives. However, more parameters mean more complex correlation patterns. Although the training load variation pattern associated with injury risk was identified using the SHAP approach, an outstanding question remains: how does this change pattern affect injury risk (e.g., a “dose-response pattern”)? Since the current research evidence and prior knowledge remain insufficient to provide a simplified and definitive answer to this question, it must be investigated in further studies. According to Bittencourt et al. (2016), the limitation of the “explanatory power” of complex phenomena should not prevent us from trying to improve the “predictive power” of injury occurrence. Therefore, applying this model in training practice is believed to reduce the incidence of injury in runners effectively.

### 4.4 Practical applications and limitations

This study proposed an injury risk prediction model based on time-series image coding and deep learning algorithms with sensitivity and specificity to runners’ injury risk. It is important to note that the variables used in this study are primarily available from wearable device recordings, which meant that integrating the method into a wearable device analysis platform would help manage runners’ injury risk. However, there are still several limitations to this study. Firstly, the ability to predict injury risk using quantitative training load indicators is limited. Injury risk in the real world results from a combination of factors, and there is a limit to the amount of information on injury risk that training load monitoring can provide. Secondly, our model has limitations in its interpretability. Ideally, a predictive model for assessing the risk of injury should not only exhibit high precision but also offer a level of interpretability. This attribute would greatly assist coaches and team physicians in formulating effective training programs and implementing targeted intervention strategies. In the future, we will investigate the interpretability of the model using methods such as knowledge distillation.

## 5 Conclusion

This study proposes an injury risk prediction model for runners based on time-series image encoding and deep learning that automatically extracts information about injury risk patterns. Compared to models reported in the literature, this approach performs better in identifying injuries, confirming the applicability of this modeling approach in the assessment of sports injury risk. In addition, through attribution analysis of the model, this study found that training volume is a significant predictor of runner injury risk and discovered the potentially high-dimensional and complex pattern of association between training load and injury risk. However, due to the limitations of *a priori* knowledge, this complex relationship has not been fully revealed and further research is still needed. Nevertheless, given the excellent discriminability, robustness, and generality of the model proposed in this study, it can be applied to injury prevention practice and provide a new analytical method for future injury prevention research.

## Data Availability

Publicly available datasets were analyzed in this study. This data can be found here: https://www.kaggle.com/datasets/shashwatwork/injury-prediction-for-competitive-runners.
