# Corrigendum: A novel approach for sports injury risk prediction: based on time-series image encoding and deep learning

**DOI:** 10.3389/fphys.2024.1441107

**Published:** 2024-07-22

**Authors:** Xiaohong Ye, Yuanqi Huang, Zhanshuang Bai, Yukun Wang

**Affiliations:** ^1^ Chengyi College, Jimei University, Xiamen, China; ^2^ School of Physical Education and Sport Science, Fujian Normal University, Fuzhou, China; ^3^ School of Tourism and Sports Health, Hezhou University, Hezhou, China; ^4^ Institute for Sport Business, Loughborough University London, London, United Kingdom

**Keywords:** injury prevention, deep learning, time series, injury risk pattern, injury risk prediction

In the published article, there was an error in **Materials and methods**, *Data processing*, paragraph five. A correction has been made to the penultimate sentence, which previously stated: “The sampling ratio is set according to the sample imbalance rate.”

The corrected sentence appears below:

“The sampling ratio is set according to the sample balance rate.”

In the published article, there was an error in **Materials and methods**, *Model architecture*. A correction has been made to the fourth sentence of paragraph two and the ninth sentence of paragraph four. These sentences previously stated: “The initial learning rate of the adadelta optimizer was set to 0.01.”

The corrected sentence appears below:

“The initial learning rate of the adadelta optimizer was set to 1.0 with a decay rate of 0.95.”

In the published article, there was an error in **Materials and methods**, *Model architecture,* paragraph five. A correction has been made to the penultimate sentence, which previously stated: “This study set α to 0.55 and γ to 5.”

The corrected sentence appears below:

“This study’s tuning process for α and γ was based on empirical. The α for the optimal model was approximately 0.986 (i.e., 1 minus the ratio of minority samples to the total sample), while γ was set to 3.5.”

The authors apologize for these errors and state that this does not change the scientific conclusions of the article in any way. The original article has been updated.

