# Supplementary material for: A novel approach for sports injury risk prediction: based on time-series image encoding and deep learning
Source: Front Physiol. 2023 Dec 18;14:1174525. doi: 10.3389/fphys.2023.1174525 (PMC10773721; doi:10.3389/fphys.2023.1174525)
Supplement: Supplementary file 1 [file DataSheet1.PDF]

In [1]:

```python
import pandas as pd
import numpy as np
import time

Train = pd.read_excel("TrainSet.xlsx")
Train.info()
```

```
<class 'pandas.core.frame.DataFrame'>
RangeIndex: 39722 entries, 0 to 39721
Data columns (total 73 columns):
 #   Column                         Non-Null Count  Dtype
---  ------                         --------------  -----
 0   nr. sessions                   39722 non-null  int64
 1   total km                       39722 non-null  float64
 2   km Z3-4                        39722 non-null  float64
 3   km Z5-T1-T2                    39722 non-null  float64
 4   km sprinting                   39722 non-null  float64
 5   strength training              39722 non-null  int64
 6   hours alternative              39722 non-null  float64
 7   perceived exertion             39722 non-null  float64
 8   perceived trainingSuccess      39722 non-null  float64
 9   perceived recovery             39722 non-null  float64
 10  nr. sessions.1                 39722 non-null  int64
 11  total km.1                     39722 non-null  float64
 12  km Z3-4.1                      39722 non-null  float64
 13  km Z5-T1-T2.1                  39722 non-null  float64
```

In [2]:

```python
Train.head(10)
```

Out[2]:

	nr. sessions	total km	km Z3-4	km Z5-T1-T2	km sprinting	strength training	hours alternative	perceived exertion	perceived trainingSuccess	per re
**0**	1	5.8	0.0	0.6	1.2	0	0.00	0.11	0.00	
**1**	0	0.0	0.0	0.0	0.0	0	0.00	-0.01	-0.01	
**2**	1	0.0	0.0	0.0	0.0	1	0.00	0.10	0.00	
**3**	0	0.0	0.0	0.0	0.0	0	0.00	-0.01	-0.01	
**4**	1	0.0	0.0	0.0	0.0	0	1.08	0.08	0.00	
**5**	1	16.4	10.0	0.0	0.0	1	0.00	0.11	0.00	
**6**	1	0.0	0.0	0.0	0.0	0	1.00	0.10	0.00	
**7**	1	5.2	0.0	0.5	1.2	0	0.00	0.10	0.00	
**8**	0	0.0	0.0	0.0	0.0	0	0.00	-0.01	-0.01	
**9**	1	0.0	0.0	0.0	0.0	1	0.00	0.10	0.00	

10 rows × 73 columns

In [3]:

```
Train.describe()
```

Out[3]:

	nr. sessions	total km	km Z3-4	km Z5-T1-T2	km sprinting	strength training
**count**	39722.000000	39722.000000	39722.000000	39722.000000	39722.000000	39722.000000
**mean**	0.832838	6.991433	0.677139	0.585399	0.076119	0.117466
**std**	0.581688	7.404181	2.257641	1.818250	0.494930	0.327637
**min**	0.000000	0.000000	0.000000	0.000000	0.000000	0.000000
**25%**	0.000000	0.000000	0.000000	0.000000	0.000000	0.000000
**50%**	1.000000	6.000000	0.000000	0.000000	0.000000	0.000000
**75%**	1.000000	12.000000	0.000000	0.000000	0.000000	0.000000
**max**	2.000000	55.900000	42.200000	48.000000	40.000000	2.000000

8 rows × 73 columns

Data cleaning

In [4]:

```
import numba

# Clear invalid data: Exclude the data that has not experienced any sports training for 7 da
@numba.njit(parallel=True)
def judge_sum(a, b, c, d, e, f, g):
    return a + b + c + d + e + f + g

def DataClear(data):
    columns_original = data.columns
    data_original = data.values
    judge = judge_sum(data_original[:, 0], data_original[:, 10], data_original[:, 20], data_ori
                      data_original[:, 40], data_original[:, 50], data_original[:, 60])

    index = np.where(judge!=0)
    data_new = data_original[index]
    return pd.DataFrame(data_new, columns=columns_original)
```

In [5]:

```
import time

start = time.time()

Train_Clear = DataClear(Train)

end = time.time()
print("Train samples:", Train_Clear.shape[0],"time:", end-start)
```

Train samples: 37629 time: 0.8131198883056641

In [6]:

```
Train_Clear.describe()
```

Out[6]:

	nr. sessions	total km	km Z3-4	km Z5-T1-T2	km sprinting	strength training
**count**	37629.000000	37629.000000	37629.000000	37629.000000	37629.000000	37629.000000
**mean**	0.879162	7.380310	0.714802	0.617960	0.080353	0.124000
**std**	0.562543	7.416278	2.313770	1.862741	0.508173	0.335421
**min**	0.000000	0.000000	0.000000	0.000000	0.000000	0.000000
**25%**	1.000000	0.000000	0.000000	0.000000	0.000000	0.000000
**50%**	1.000000	6.800000	0.000000	0.000000	0.000000	0.000000
**75%**	1.000000	12.300000	0.000000	0.000000	0.000000	0.000000
**max**	2.000000	55.900000	42.200000	48.000000	40.000000	2.000000

8 rows × 73 columns

In [7]:

```
def Padding(data):
    day_7 = data[0:10]
    day_6 = data[10:20]
    day_5 = data[20:30]
    day_4 = data[30:40]
    day_3 = data[40:50]
    day_2 = data[50:60]
    day_1 = data[60:70]
    day_8 = np.zeros(10,dtype=float) # padding
    data_reshape = np.vstack((day_1,day_2,day_3,day_4,day_5,day_6,day_7,day_8))

    return data_reshape

@numba.njit()
def TransformInt(data):
    return int(data)

def List_to_arr(data_list, data):
    new_data = []
    data_information = []

    for i in range(len(data_list)):
        temp_feature = data_list[i]
        temp_information = data[i]
        if np.sum(temp_feature[:,0]) != 0:

            new_data.append(temp_feature)
            data_information.append(temp_information)

    new_data = np.array(new_data)
    data_information = np.array(data_information)
    ID = data_information[:,0]
    Label = np.array(list(map(TransformInt,data_information[:,1])))
    Date = data_information[:,2]

    return new_data, ID, Label, Date


```

In [8]:

```
import time

start = time.time()

Train_reshape = list(map(Padding, Train_Clear.values))
Train_Feature, Train_ID, Train_Label, Train_Date = List_to_arr(Train_reshape,Train_Clear.val

end = time.time()

print("time:",end-start,
      f"\n Original TrainSet: {np.array(Train_reshape).shape}",
      )
```

```
time: 0.8144659996032715
 Original TrainSet: (37629, 8, 10)
```

Gramian Angular Field

In [9]:

```
n_sample = 37133
```

The original time series

In [32]:

```
import matplotlib.pyplot as plt

X_new = Train_Feature[n_sample,:,1] # With "total km" for example
X_sample = [list(range(0, 8, 1)), X_new]

fig = plt.figure(figsize=(3,2),dpi=300,edgecolor='black')
plt.plot(X_sample[0], X_sample[1])

plt.xlabel("day")
plt.ylabel("total km")
plt.tight_layout()
plt.show()
```

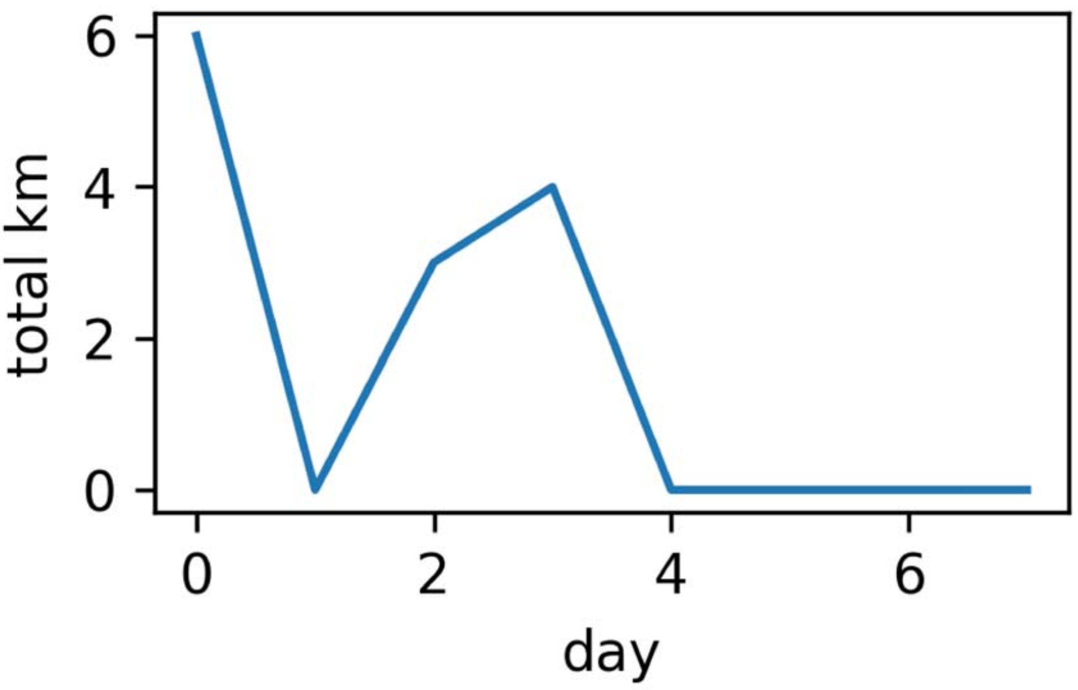

In [33]:

```
#fig.savefig("C:\\Users\\47762\\Desktop\\Figure\\original time series.jpg")
```

Time series after normalization

In [34]:

```
from pyts.preprocessing import MinMaxScaler

# normalization
scaler = MinMaxScaler()
X_sample_scaler = scaler.transform(X_sample)

fig = plt.figure(figsize=(3,2),dpi=300,edgecolor='black')
plt.plot(X_sample_scaler[0,:], X_sample_scaler[1,:])
plt.xlabel("timestamp")
plt.ylabel("total km")
plt.tight_layout()
plt.show()
```

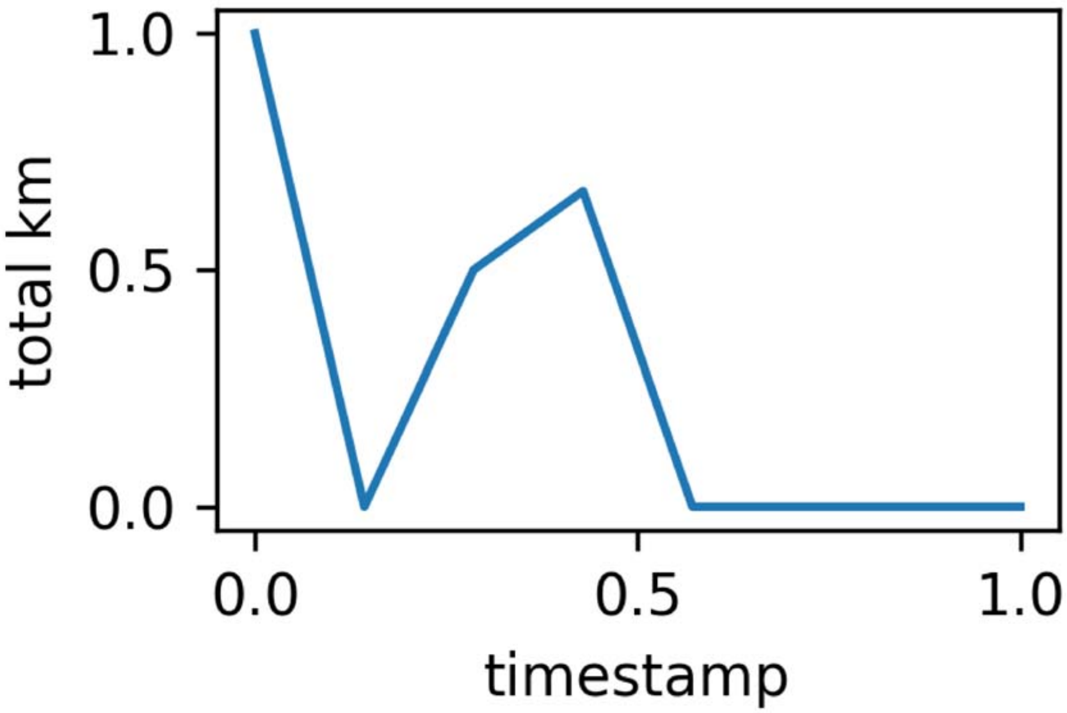

In [35]:

```
#fig.savefig("C:\\Users\\47762\\Desktop\\Figure\\scaler time series.jpg")
```

Polar coordinate system

In [36]:

```
arccos_X = np.arccos(X_sample_scaler[1,:])
fig, ax = plt.subplots(figsize=(6,4),dpi=300,subplot_kw={'projection': 'polar'})
ax.plot(X_sample[0], arccos_X)
ax.set_rmax(2)
ax.set_rticks([0.5, 1, 1.5, 2]) # Less radial ticks
ax.set_rlabel_position(-22.5) # Move radial labels away from plotted line
ax.grid(True)
ax.set_title("Polar coordinates", va='bottom')
plt.tight_layout()
plt.show()
```

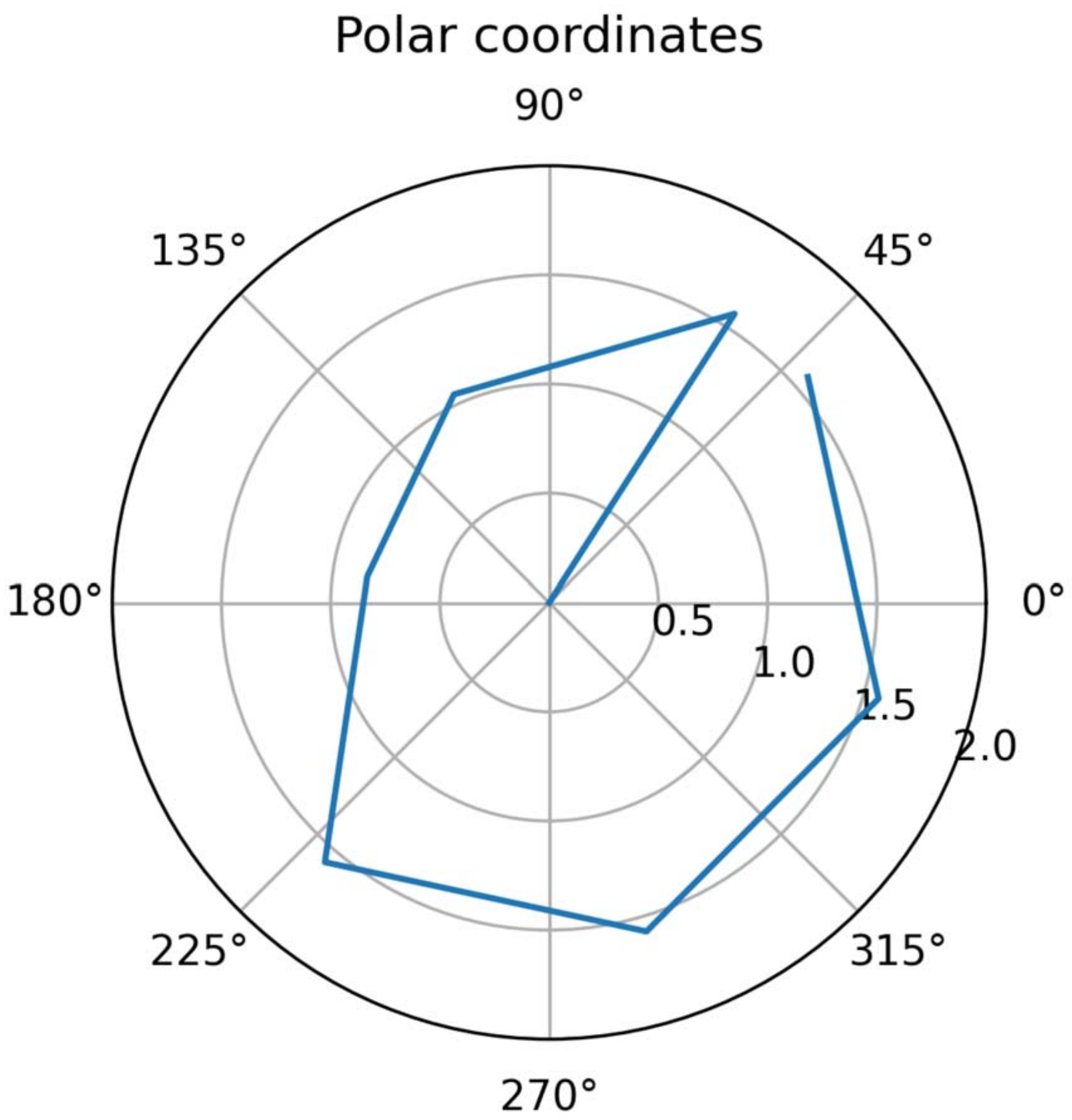

In [37]:

```
#fig.savefig("C:\\Users\\47762\\Desktop\\Figure\\coordinates time series.jpg")
```

Gramian Angular Summation Field

In [38]:

```
from matplotlib.pyplot import MultipleLocator

fig = plt.figure(figsize=(3,2),dpi=300,edgecolor='black')
field = [a+b for a in arccos_X for b in arccos_X]
gram = np.cos(field).reshape(8,8)
plt.imshow(pd.DataFrame(gram ).rename(index={0:1, 1:2, 2:3, 3:4, 4:5, 5:6, 6:7, 7:8},
                                       columns={0:1, 1:2, 2:3, 3:4, 4:5, 5:6, 6:7, 7:8}
ax = plt.gca()
major_locator=MultipleLocator(1)
ax.xaxis.set_major_locator(major_locator)
ax.yaxis.set_major_locator(major_locator)
plt.tight_layout()
plt.colorbar()
```

Out[38]:

<matplotlib.colorbar.Colorbar at 0x19d771d1f60>

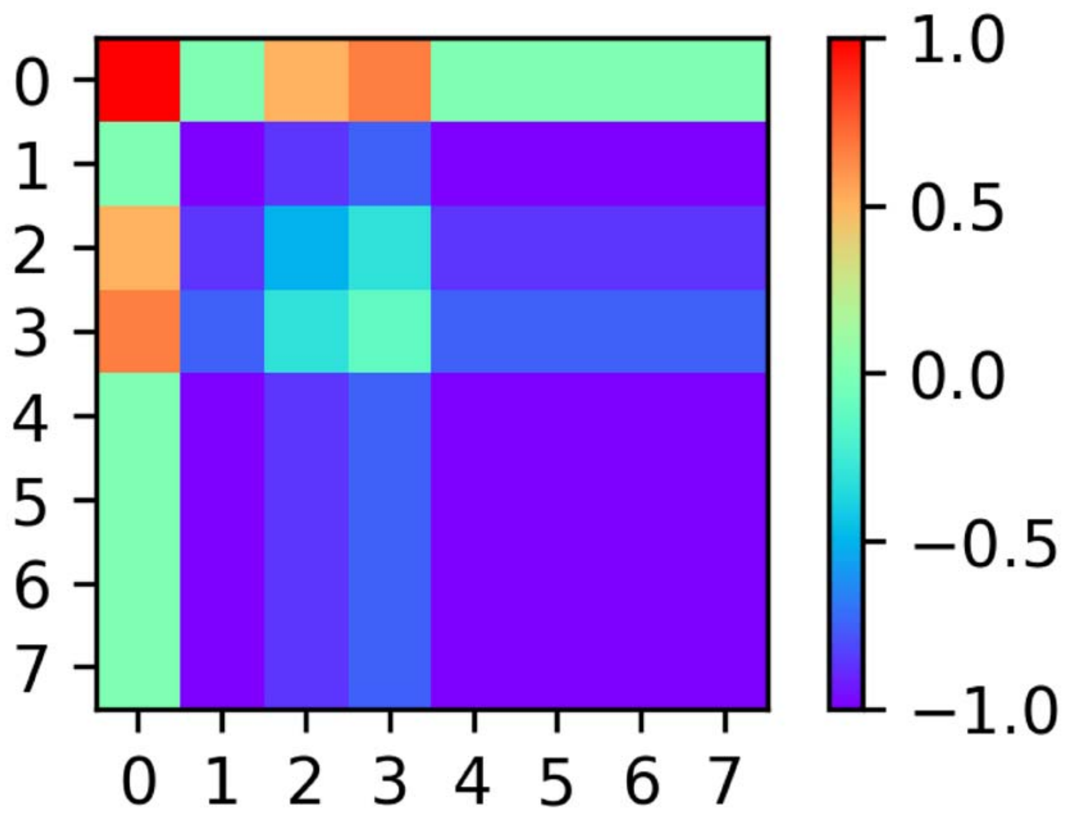

In [39]:

```
#fig.savefig("C:\\Users\\47762\\Desktop\\Figure\\GASF.jpg")
```

Gramian Angular Differential Field

In [40]:

```python
fig = plt.figure(figsize=(3,2),dpi=300,edgecolor='black')
field = [a-b for a in arccos_X for b in arccos_X]
gram = np.sin(field).reshape(8,8)
plt.imshow(pd.DataFrame(gram ).rename(index={0:1, 1:2, 2:3, 3:4, 4:5, 5:6, 6:7, 7:8},
                                        columns={0:1, 1:2, 2:3, 3:4, 4:5, 5:6, 6:7, 7:8}
ax = plt.gca()
major_locator=MultipleLocator(1)
ax.xaxis.set_major_locator(major_locator)
ax.yaxis.set_major_locator(major_locator)
plt.tight_layout()
plt.imshow(gram, cmap='rainbow')
plt.colorbar()
```

Out[40]:

```
<matplotlib.colorbar.Colorbar at 0x19d78260748>
```

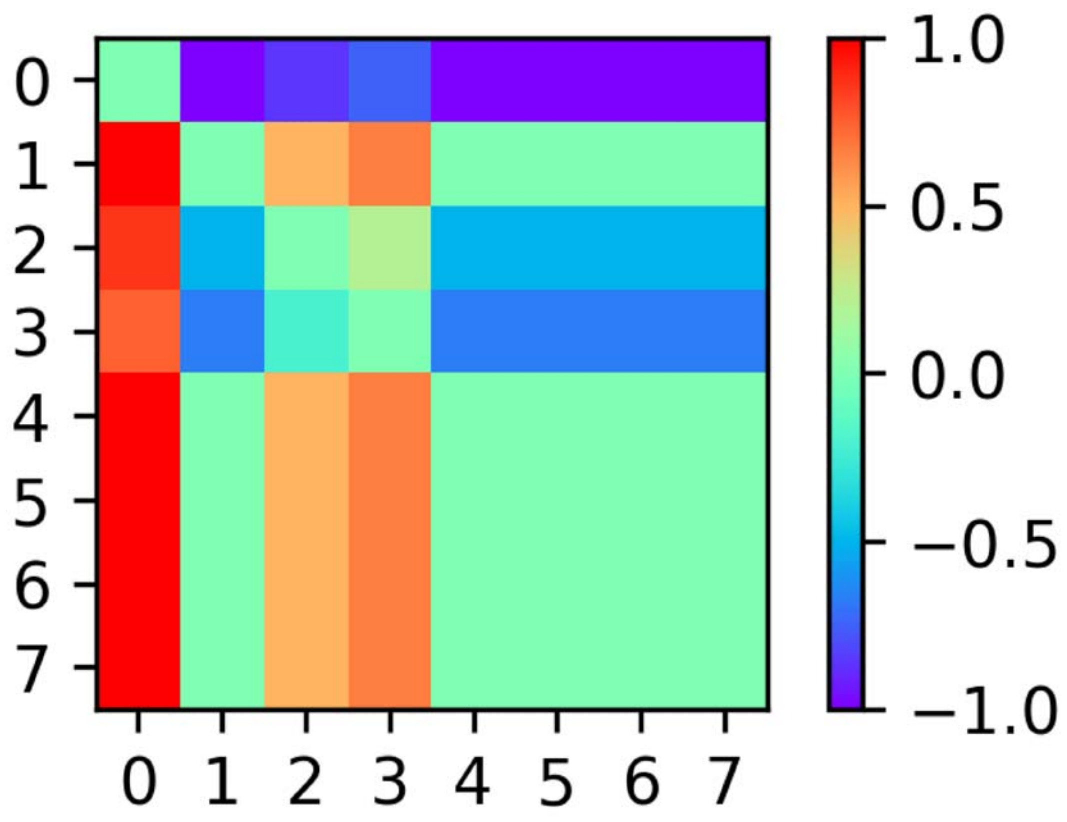

In [41]:

```python
#fig.savefig("C:\\Users\\47762\\Desktop\\Figure\\GADF.jpg")
```

Markov Transition field Transformation

In [42]:

```
X_new = X_sample_scaler[1,:]
```

In [43]:

```
from pyts.preprocessing.discretizer import KBinsDiscretizer

n_bins = 5
strategy = 'quantile'
discretizer = KBinsDiscretizer(n_bins = n_bins, strategy = strategy, raise_warning = False)
X = X_new.reshape(1, -1)
X_disc = discretizer.fit_transform(X)[0]
X_disc
```

Out[43]:

```
array([3, 0, 2, 3, 0, 0, 0, 0], dtype=int64)
```

In [44]:

```
m_adj = np.zeros((n_bins,n_bins))
for k in range(len(X_disc) - 1):
    # matrix iteration
    index = X_disc[k]
    next_index = X_disc[k+1]
    m_adj[next_index][index] += 1

print(m_adj)
```

```
[[3. 0. 0. 2. 0.]
 [0. 0. 0. 0. 0.]
 [1. 0. 0. 0. 0.]
 [0. 0. 1. 0. 0.]
 [0. 0. 0. 0. 0.]]
```

In [45]:

```
mtm = m_adj/m_adj.sum(axis=0)
print(mtm)
```

```
[[0.75  nan 0.   1.    nan]
 [0.    nan 0.   0.    nan]
 [0.25  nan 0.   0.    nan]
 [0.    nan 1.   0.    nan]
 [0.    nan 0.   0.    nan]]

D:\Anaconda3\lib\site-packages\ipykernel_launcher.py:1: RuntimeWarning: invalid v
alue encountered in true_divide
  """Entry point for launching an IPython kernel.
```

In [46]:

```
n_t = len(X_disc)
mtf = np.zeros((n_t, n_t))

for i in range(n_t):
    for j in range(n_t):
        mtf[i, j] = mtm[X_disc[i]][X_disc[j]]*100
```

In [47]:

```
mtf
```

Out[47]:

```
array([[  0.,   0., 100.,   0.,   0.,   0.,   0.,   0.],
       [100.,  75.,   0., 100.,  75.,  75.,  75.,  75.],
       [  0.,  25.,   0.,   0.,  25.,  25.,  25.,  25.],
       [  0.,   0., 100.,   0.,   0.,   0.,   0.,   0.],
       [100.,  75.,   0., 100.,  75.,  75.,  75.,  75.],
       [100.,  75.,   0., 100.,  75.,  75.,  75.,  75.],
       [100.,  75.,   0., 100.,  75.,  75.,  75.,  75.],
       [100.,  75.,   0., 100.,  75.,  75.,  75.,  75.]])
```

In [48]:

```
fig = plt.figure(figsize=(3,2),dpi=300,edgecolor='black')
plt.imshow(pd.DataFrame(mtf ).rename(index={0:1, 1:2, 2:3, 3:4, 4:5, 5:6, 6:7, 7:8},
                                              columns={0:1, 1:2, 2:3, 3:4, 4:5, 5:6, 6:7, 7:8}
ax = plt.gca()
major_locator=MultipleLocator(1)
ax.xaxis.set_major_locator(major_locator)
ax.yaxis.set_major_locator(major_locator)
plt.tight_layout()
plt.colorbar()
plt.show()
```

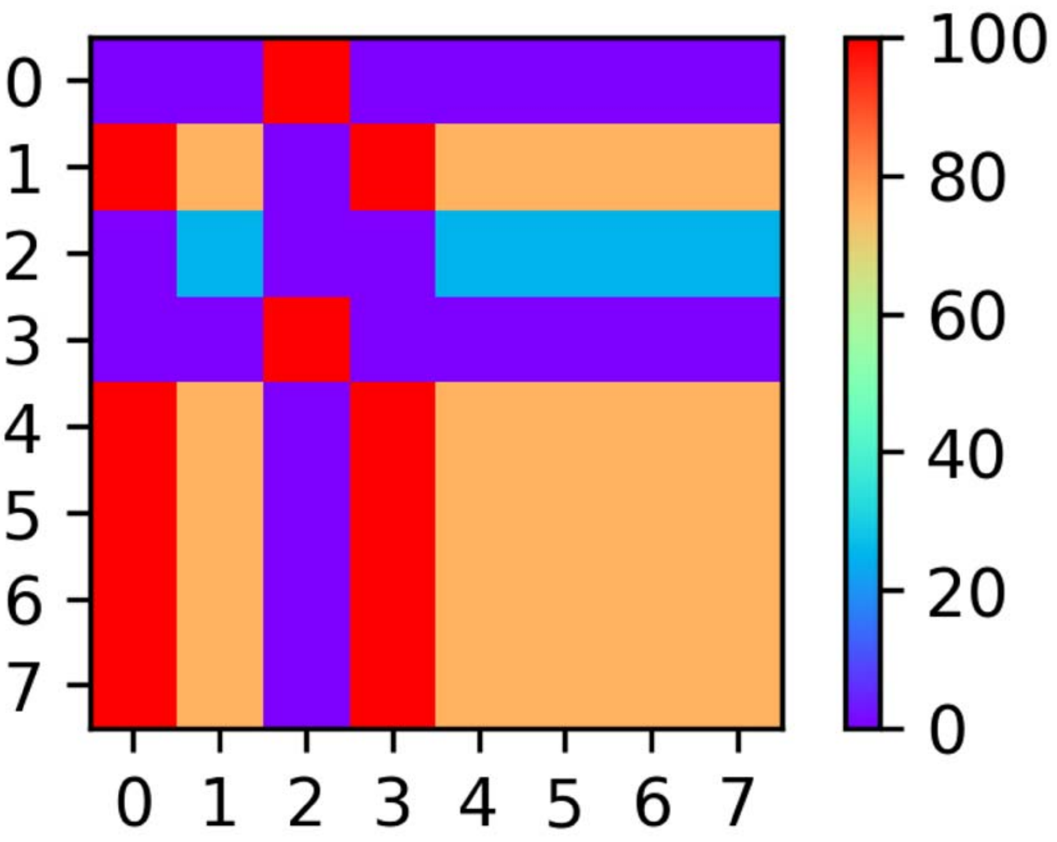

In [49]:

```
#fig.savefig("C:\\Users\\47762\\Desktop\\Figure\\MTF.jpg")
```

Recurrence Plot Transformation

In [50]:

```
from scipy.spatial.distance import pdist, squareform

eps=0.10
steps=10
d = pdist(X_new[:,None])
d = np.floor(d/eps)
d[d>steps] = steps
Z = squareform(d)
d
```

Out[50]:

```
array([10.,  5.,  3., 10., 10., 10., 10.,  5.,  6.,  0.,  0.,  0.,  0.,
        1.,  5.,  5.,  5.,  5.,  6.,  6.,  6.,  6.,  0.,  0.,  0.,  0.,
        0.,  0.])
```

In [51]:

```
Z
```

Out[51]:

```
array([[ 0., 10.,  5.,  3., 10., 10., 10., 10.],
       [10.,  0.,  5.,  6.,  0.,  0.,  0.,  0.],
       [ 5.,  5.,  0.,  1.,  5.,  5.,  5.,  5.],
       [ 3.,  6.,  1.,  0.,  6.,  6.,  6.,  6.],
       [10.,  0.,  5.,  6.,  0.,  0.,  0.,  0.],
       [10.,  0.,  5.,  6.,  0.,  0.,  0.,  0.],
       [10.,  0.,  5.,  6.,  0.,  0.,  0.,  0.],
       [10.,  0.,  5.,  6.,  0.,  0.,  0.,  0.]])
```

In [52]:

```
fig = plt.figure(figsize=(3,2),dpi=300,edgecolor='black')
plt.imshow(pd.DataFrame(Z ).rename(index={0:1, 1:2, 2:3, 3:4, 4:5, 5:6, 6:7, 7:8},
                                                    columns={0:1, 1:2, 2:3, 3:4, 4:5, 5:6, 6:7, 7:8}
ax = plt.gca()
major_locator=MultipleLocator(1)
ax.xaxis.set_major_locator(major_locator)
ax.yaxis.set_major_locator(major_locator)
plt.tight_layout()
plt.colorbar()
plt.show()
```

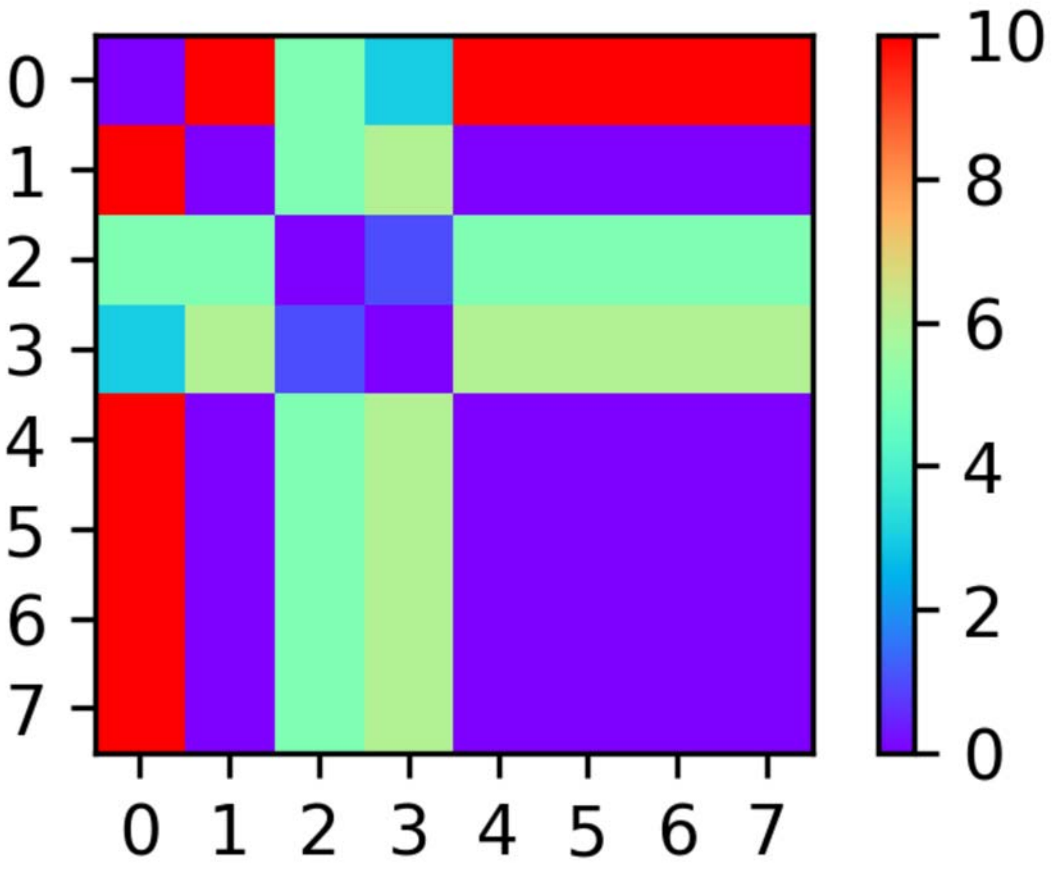

In [31]:

```
#fig.savefig("C:\\Users\\47762\\Desktop\\Figure\\RP.jpg")
```

In [ ]:

```

```
